# Public health ethics: critiques of the “new normal”

**DOI:** 10.1007/s40592-022-00163-7

**Published:** 2022-09-27

**Authors:** Euzebiusz Jamrozik

**Affiliations:** 1 The Ethox Centre & Wellcome Centre for Ethics and Humanities, University of Oxford, Old Rd, OX3 7LF Headington, Oxford, UK; 2 Monash Bioethics Centre, Monash University, Melbourne, Australia; 3 Department of Medicine, Royal Melbourne Hospital, University of Melbourne, Melbourne, Australia

## Abstract

The global response to the recent coronavirus pandemic has revealed an ethical crisis in public health. This article analyses key pandemic public health policies in light of widely accepted ethical principles: the need for evidence, the least restrictive/harmful alternative, proportionality, equity, reciprocity, due legal process, and transparency. Many policies would be considered unacceptable according to pre-pandemic norms of public health ethics. There are thus significant opportunities to develop more ethical responses to future pandemics. This paper serves as the introduction to this Special Issue of Monash Bioethics Review and provides background for the other articles in this collection.

## Introduction

Donald Henderson (1928–2016) was a prominent medical epidemiologist who, among other achievements, led the successful World Health Organization (WHO) smallpox eradication campaign. In a 2016 paper on optimal public health responses to influenza pandemics, Henderson and co-authors identify an “Overriding Principle” (Inglesby, Nuzzo et al. 2006):

[C]ommunities faced with epidemics … respond best and with the least anxiety when the normal social functioning of the community is least disrupted

Many global responses to the recent coronavirus pandemic, including many of Australia’s policy responses, have failed to follow not only this “Overriding Principle” but also many other principles of public health ethics (Table 1) (Childress, Faden et al. 2002, Upshur 2002, Selgelid 2009, Jamrozik and Selgelid 2019). The “new normal” of police-enforced lockdowns, border closures with bans on citizens leaving and returning home, prolonged school closures, and, in general, restrictive public health policies with large penalties for non-compliance, diverges from the “old normal” of public health pandemic response planning, which generally recommended against widespread, prolonged, and/or punitive policies because the harms of such policies would likely outweigh any benefits in terms of reduced infectious disease transmission (and the harms would often be inequitably distributed). While extreme measures such as school or business closures were previously recommended for high severity pandemics at the peak of initial waves, these measures were to be implemented only for the shortest possible time (i.e., a maximum of several weeks). Most pandemic plans were also predicated on the reliable assumption that there is no sustainable way to eliminate pandemic viruses (since, among other things, there is no realistic expectation of developing a vaccine capable of this feat). As a result, for example, a 2019 World Health Organization report on non-pharmaceutical interventions (NPIs) during influenza pandemics recommended *against* the following measures "*under any circumstances*", i.e., no matter how severe the pandemic threat. Use of contact tracing (since this is costly and often futile for a widespread or rapidly spreading infection), mandatory quarantine of exposed individuals (since almost every individual will sooner or later exposed to a pandemic virus), entry and exit screening (since this has been shown to be ineffective), and border closures (since these impose large costs, are often discriminatory, and involve excessive restriction of citizens’ rights) (WHO 2019). In parallel, however, some pandemic planning activities in the early 21st Century had increasingly recommended the use of police power and coercive control in public health responses, despite the tendency for such securitised approaches to be discriminatory, non-transparent, and the likelihood that excessive use of coercion would undermine trust in public health (ACLU 2019). The coronavirus disease 2019 (covid19) pandemic provided a challenge to public health agencies to strike a reasonable balance between key ethical values - in other words, to promote health while avoiding causing harm, exacerbating inequality, or using excessive coercion. This article summarises some ethical aspects of non-pharmaceutical covid19 pandemic policy responses with a particular focus on Australia. It also provides background for the papers in this special issue which include further ethical, philosophical, and economic analyses of pandemic public health policies. While the predominant focus of this issue is on non-pharmaceutical interventions (NPIs) policy regarding pharmaceutical interventions such as vaccines also raises a range of ethical issues that have received significant attention elsewhere (Gur-Arie, Jamrozik et al. 2021, Bardosh, de Figueiredo et al. 2022, Giubilini, Savulescu et al. 2022, Kraaijeveld, Gur-Arie et al. 2022).

**Table 1:** Principles of public health ethics

Public Health Ethics Principle	Interpretation/Example
Need for evidence	Evidence of likely benefits is needed to justify imposition of potentially burdensome public health interventions.
Least restrictive alternative	Where two interventions are expected to be equally effective, the intervention that involves the least restrictions of liberty should be selected.
Least harmful alternative	Where two interventions are expected to be equally effective, the intervention that involves the least harms should be selected.
Proportionality	The burdens (and/or harms) involved in an intervention should be outweighed by public health benefits achieved.
Equity	The intervention should be implemented (and burdens imposed) in an equitable, non-discrimintory manner.
Reciprocity	Those who benefit from public health policies/interventions have a reciprocal duty to assist and/or compensate those on whom burdens are imposed.
Due legal process	Appropriate legal procedures should be followed and individuals should have the right of appeal.
Transparency	Policymaking should be transparent and democratic.

* Adapted from (Jamrozik and Selgelid 2019)

## Public health ethics

The ethical justification of public health intervention requires more than just the expectation that an intervention will produce a (net) improvement public health (over and above the harms of the intervention). In addition to *health*, two other (sets of) values are key to the justification of public health policy: *fairness*, e.g., regarding the distribution of benefits and harms of an intervention in a population, and *freedom*, e.g., to move and interact with others without unjustified externally-imposed restrictions (Selgelid 2009) [Table 2]. These plain language terms are used here to refer to three key sets or families of values that are widely held to be intrinsically important. While public health interventions typically aim to create or promote a particular public health benefit (or avert a particular harm), all interventions also have costs and some have harmful (as well as beneficial) effects, including on human health. In addition, interventions can increase or decrease fairness within society (e.g., in terms of the distribution of health outcomes) and can infringe on individual freedoms to a greater or lesser degree. Insofar as each of these values carries moral weight, policies that involve harm, increase unfairness, or infringe on individual freedoms require ethical justification(Selgelid 2009).

**Table 2:** Key values in public health ethics

Value	Other relevant values or concepts
Health	Utility, wellbeing, benefits, harms, etc.
Fairness	Equality, equity, distributive justice, procedural justice, etc.
Freedom	Liberty, autonomy, human rights, respect for persons, etc.

The principles of public health ethics [Table 1] can ideally help public health agencies and policymakers to strike a reasonable balance between key values [Table 2]. Used well, these principles can be used to determine the extent to which a given policy would be ethically justifiable. Such ethical evaluations also require reviewing the best available empirical evidence regarding the expected benefits and harms (including harms or burdens related to liberty restrictions) involved in a policy, as well as the likely distribution of these outcomes in the community.

## Evaluating the ethical acceptability of public health measures over time

Public health responses to a pandemic should arguably be sustainable, based on the expectation that pandemic viruses will become endemic and cause disease for many years(Heriot and Jamrozik 2021). For example, the virus that caused the 1918 influenza pandemic circulated continuously for 40 years until 1957 (Taubenberger and Morens 2006).

While extraordinary measures might have been ethically justifiable for a short period of time at the start of the current coronavirus pandemic in the face of (some degree of) uncertainty about the risks posed by the pathogen, the prolonged use of stringent or harmful measures is and was often unjustifiable. Indeed, there are sometimes strong moral reasons to relax stringent measures when it becomes clear (or likely) that they are non-beneficial, offer net harms, involve excessive liberty restriction, or increase unfairness in society (e.g., by primarily benefitting people who are already well off or primarily harming those who are badly off). While “two weeks” of lockdown i.e., quasi-universal coercive social distancing measures, might have been considered justifiable at the peak of initial waves of illness and hospitalisation, prolonged lockdowns created enormous cumulative harms that likely (especially after the availability of vaccines) outweighed their benefits on a range of reasonable weightings (Pak, Adegboye et al. 2021, Lally 2022, Lawford-Smith 2022). The remainder of this article considers the extent to which certain policies for covid19 were aligned with or diverged from the above principles of public health ethics, and identifies areas where reform might improve the ethical acceptability of responses to future pandemics.

## Need for evidence

The ethical use of public health powers is contingent on there being evidence that (i) there is a serious risk to public health and (ii) that a given intervention is likely to significantly reduce that risk without producing greater harms. In terms of a serious risk to public health, data from China in early 2020 provided clear evidence that covid19 was a major risk to older adults but was associated with very low, perhaps minimal, risks to young adults and children (The Novel Coronavirus Pneumonia Emergency Response Epidemiology Team 2020). By April 2020, Chinese data also showed that viral transmission was concentrated in indoor settings, with reports of outdoor transmission being extremely rare(Qian, Miao et al. 2021). These epidemiological features were repeatedly confirmed in other settings (Salje, Kiem et al. 2020, Spiegelhalter 2020, Bulfone, Malekinejad et al. 2021). The risks^1^ of covid19 to young healthy people (especially children) remain low, with older adults, especially those with comorbidities, being over 1000 times more likely to suffer severe or fatal covid19(Spiegelhalter 2020). At the time of writing (August 2022), zero cases of outdoor transmission confirmed by phylogenetic sequencing of the virus have been published in peer-reviewed scientific literature, although one case with sequencing has been reported by Australian health authorities^2^. Given this evidence, one might think that public health interventions (especially burdensome or restrictive interventions) for this disease should be focused on older adults and indoor settings. Instead, many jurisdictions opted for multiple quasi-universal mandatory interventions (even among young people and/or in outdoor settings) resulting in extreme disruptions of normal social functioning.

Non-pharmaceutical interventions (NPIs) for respiratory viruses have rarely been tested in rigorous studies (e.g., randomized controlled trials). However, prior to the current pandemic, there was randomized controlled trial evidence that, for example, household quarantine could reduce workplace transmission of respiratory viruses at the cost of increasing transmission within households subject to quarantine(Miyaki, Sakurazawa et al. 2011). There was also evidence that cloth face masks were ineffective (MacIntyre, Seale et al. 2015) (on the basis of which authorities initially recommended against their use for covid19 (World Health Organization 2020)) and that, more generally, widespread community masking provided little or no reduction in the transmission of respiratory viruses(Jefferson, Del Mar et al. 2020).

There is always some uncertainty about the extent to which evidence from one virus or population will be generalisable to another. However, this should not lead to radical scepticism, discarding of previous evidence, or *carte blanche* for public health agencies to institute any measure no matter how weak its evidence base. Instead, while public health authorities might reasonably implement measures (for a novel virus) that had weak or no effect on other similar diseases in the initial response, there is arguably an ethical imperative that such policies be coupled with a plan to collect evidence (ideally using randomized and/or controlled study designs) regarding the resultant benefits and harms. There is an especially strong ethical rationale to collect high quality data when interventions are mandated, since the enforced liberty restrictions involved in mandates arguably require more ethical (and thus evidentiary) justification. It is therefore disappointing that so few high-quality studies of NPIs have been conducted during the pandemic to date. While some might claim that it would be unethical to conduct trials of NPIs (e.g., masks), such claims presumably rest on a belief that the benefits of a given intervention clearly outweigh the harms even without conducting a trial to produce such data. Yet without controlled data to support beliefs in the effectiveness, cost-effectiveness, or harmlessness of an intervention, it is hard to justify a high degree of epistemic confidence in the claim that benefits clearly outweigh harms or that a trial would be unethical. To date, for example, only two randomized controlled trials have studied the effectiveness of public masking for covid19. Consistent with pre-pandemic evidence for other viruses, these trials found that masks were associated with small or no significant benefits, and that cloth masks in particular provided no benefit(Bundgaard, Bundgaard et al. 2021, Abaluck, Kwong et al. 2022).

**Fig. 1:**
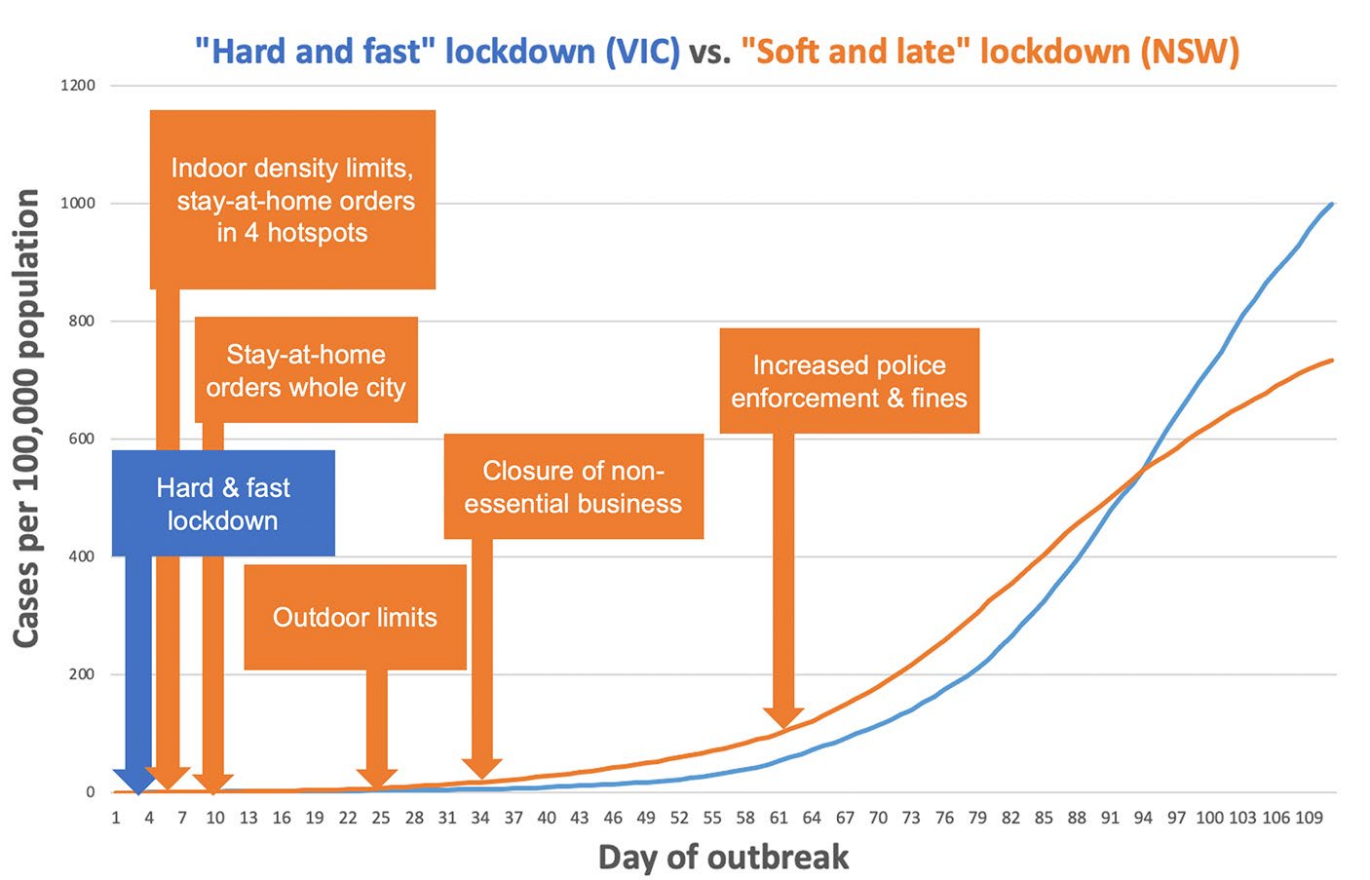
Natural experiment on the effectiveness of “hard and fast” lockdown. In mid-2021, the Australian states of Victoria (VIC) and New South Wales (NSW) faced similar outbreaks of a similar variant of covid19 at similar times. Victoria instituted an immediate hard lockdown and experienced a higher peak of cases of covid19, whereas New South Wales gradually instituted a range of measures over several weaks and a experienced a lower peak of cases. Data available at https://www.covid19data.com.au/compare-lockdowns [accessed 1 September 2022].

Some large scale “natural experiments” nevertheless provide insight into the likely effectiveness of several other NPIs. A natural experiment occurs, for example, when two otherwise similar populations facing similar epidemics adopt different public health policies. While data from such experiments may not be as reliable as those from randomized controlled trials, a difference in outcome between such similar populations can help to support or refute hypotheses regarding the benefits and harms of interventions (especially if it is thought that different interventions would produce large differences in outcomes). For example, regarding the use of curfews, there was no difference in covid19 incidence between jurisdictions in Germany that implemented a nocturnal curfew and those that did not(de Haas, Goetz et al. 2021), suggesting that such interventions do not have a large effect on covid19 transmission (in contexts where other public health measures are already in place). Regarding lockdowns, it has sometimes been claimed that early implementation of stringent lockdowns is a better (i.e., more effective) strategy than slower implementation of increasingly stringent measures. Yet a natural experiment in Australia in mid-2021 showed that this is not always the case. At similar times, Melbourne (Victoria) and Sydney (New South Wales) faced epidemics of closely related variants of covid19 and implemented different policies (Butterworth, Schurer et al. 2022): Melbourne implemented a rapid, stringent lockdown while Sydney implemented less stringent measures and introduced these more slowly, in a stepwise manner [See Figure 1]. Although many might have expected that Melbourne’s "hard and fast" lockdown would be more effective at controlling the virus, the initial growth of the epidemics was overall similar and Melbourne even experienced a higher per-capita peak in cases^3^ as well as worse mental health outcomes and a longer duration of highly coercive measures(Butterworth, Schurer et al. 2022). This might at least be taken as evidence that rapid, stringent lockdowns are not always superior to slower introduction of less stringent measures in terms of long-term transmission control. Insofar as stringent measures are associated with greater harms, this might also undermine the ethical case for strict lockdowns.

## Least restrictive alternative

The response to covid19 often involved extreme restrictions of individual liberty. Key infringements included controls on freedom of movement (including freedom to leave and return to one’s own country (Silva 2022)), freedom of association, freedom to protest, freedom of expression, free choice of occupation, free choice regarding medical (and non-pharmaceutical) interventions, and privacy (i.e., free choice regarding one’s own personal information). The term “lockdown” did not appear in pandemic planning documents pre-2020 and was primarily used in prison management – though experts in criminal justice have noted that approaches from prisons may be linked to (and perhaps even influence) wider social and political norms (Gottschalk 2015). Initial uses of “lockdown” in UK modelling documents used the term to refer to the combination of (i) mass social distancing, (ii) isolation and quarantine, (iii) school and university closure, and (iv)workplace closure(Ferguson, Laydon et al. 2020). These initial uses of “lockdown” were agnostic, one might think, about whether there would be punitive police enforcement, restrictions on outdoor activities, or bans on protest as part of lockdowns. Yet the term rapidly became associated with coercive and often punitive enforcement of such policies, including draconian restrictions of citizens’ democratic freedoms (Soutphommasane and Stears 2022). In other words, not only did lockdowns involve significant liberty restrictions, they were also often accompanied by police and military enforcement, high financial penalties for violation of public health orders (including for children), and even in some cases imprisonment for those who did not comply^4^. Legal penalties and sometimes large fines often applied even where restrictive policies produced little or no public health benefit, for example in outdoor settings, where Australian residents could be fined around $5,000 for meeting in a park. Did such policies align with the ethical principle of the least restrictive alternative? Or could public health agencies, in retrospect, have instituted less restrictive policies without a major reduction in public health benefits?

At the population level, one of the difficulties in answering such questions lies in distinguishing between the effects of (i) mandated mass population interventions (e.g., lockdowns), (ii) voluntary behaviour change in the population, and (iii) immune or environmental factors. In prominent examples such as early lockdowns in northern Italy and the United States, voluntary behaviour change produced a large fraction of the observed association between the timing of a policy being introduced and reduced transmission (Yan, Malik et al. 2021). In many cases, transmission had already peaked by the time lockdowns were implemented (Cereda, Manica et al. 2021). The net effect of these multiple causal pathways is that the contribution of lockdowns to reducing covid19 incidence will, on average, be lower than any observed association between the onset of an intervention and a reduction in transmission. Moreover, it now appears likely that shorter duration or less stringent interventions (for example,less restriction on outdoor activities, less nocturnal curfews, less school closures, or less police enforcement) may have produced similar results (Haug, Geyrhofer et al. 2020, Sharma, Mindermann et al. 2021).

At the individual level, it is now clear that 14 days of isolation and quarantine for cases and contacts was often excessive. Most otherwise healthy infected people do not remain infectious for 14 days, and polymerase chain reaction (PCR) tests remain positive sometimes for many days after a person is no longer infectious (Killingley, Mann et al. 2022), meaning that isolation based on PCR positivity will be excessively restrictive. Further, most people subject to quarantine orders due to exposure to an infectious case do not become infected. For example,the highest risk of becoming infected occurs after exposure within households, where the average infection risk among people exposed to a case of covid19 is typically estimated to be < 20% (Madewell, Yang et al. 2021). Even in this relatively high-risk group, for every 5 people subject to quarantine after such exposures, at least 4 (on average) will *not* have been infected (and those exposed in general community settings, who were also often subject to quarantine, face lower risks of infection than those exposed within households). In terms of international quarantine, the positivity rate among international travellers arriving in Australian hotel quarantine was < 1% (New South Wales Government 2021): of the more than 300,000 people subject to hotel quarantine, more than 297,000 had not been infected before or during travel to Australia. Moreover, most people exposed to covid19 who *do *become infected develop symptoms and/or a positive test within the first week, meaning that those who are still asymptomatic and testing negative by (for example) day 7 post-exposure are much less likely to have been infected and pose lower risks to the community(Public Health England 2021). Thus, quarantine periods could in many cases have been shortened, perhaps with a negative test result prior to clearance, with minimal difference in public health risk. While it might seem trivial to suggest shorter periods of isolation and quarantine, hundreds of millions of people were likely subject to such orders worldwide, meaning that millions of person-years were spent living under unnecessarily strict and/or prolonged public health orders.

In future pandemics, policymakers arguably have an ethical responsibility to use mandatory public health powers as a last resort, to relax restrictive measures as soon as it is reasonable to think that a given amount of additional liberty infringement is no longer producing significant additional public health benefits (compared with any relevant less restrictive alternative), and to consider reductions in the duration of restrictive interventions.

## Least harmful alternative

In addition to the ethical cost of liberty infringements, pandemic policies produced enormous harm. Worldwide, many of these harms have been borne by children, especially in low- and middle-income countries, with large numbers of increased deaths under 5 years of age (not caused by the coronavirus itself), millions of additional child marriages, and major disruptions to non-covid disease programs (Sick Kids Centre for Global Child Health 2021). Children in multiple countries also suffered significant learning losses due to prolonged school closures(Moscoviz and Evans 2022). Other widespread harms in children, adolescents, and/or adults include deteriorations in mental health, disruption of screening and healthcare for non-communicable diseases, and significant increases in risk factors for ill-health such as obesity and excessive alcohol consumption(Ipsos 2021). The large economic costs of non-pharmaceutical interventions also constitute harms insofar as resources could have been better directed to produce greater health or other social benefits(Lally 2022).

Even apparently innocuous interventions such as masks produce significant harm. The potential harms of mass population masking include disruption of normal verbal communication (which can be expected to harm, for example, children and adults with baseline impairments in this area), economic harms related to the billions of dollars spent on masks, and environmental harms associated with disposable masks(Torres and De-la-Torre 2021). Focusing interventions including masks in contexts where they could be expected to produce the most benefit (rather than mandating them across entire populations) could have been expected to reduce such harms.

In the case of infectious disease outcomes, the suppression of endemic pathogens also produces harm in the form of rebound epidemics (Eden, Sikazwe et al. 2022). Endemic pathogens, including SARS-CoV-2, influenza, respiratory syncytial virus, as well as many other respiratory and gastrointestinal viruses, cause periodic (often seasonal) epidemics. Ultimately, every living person with significant social contact can expect to be infected with such ubiquitous pathogens (even those living in remote areas(Heinbecker and Irvine-Jones 1928)). This also means that after a few years of life most people have some degree of immunity which will reduce the severity of future episodes of the same disease but does not provide (complete, lifelong) protection against reinfection. The suppression of transmission of a given endemic disease in a population therefore cannot itself *prevent* disease, it can only *postpone* inevitable infections to the future – a fact often missed in early claims that a short duration of non-pharmaceutical measures would prevent deaths. Prolonged suppression of transmission can result in unusually large rebound epidemics and therefore increased pressure on health systems(Eden, Sikazwe et al. 2022). It can also be difficult to predict which strains or variants of particular pathogens will be dominant at the time of a rebound epidemic (Eden, Sikazwe et al. 2022) – if a particularly virulent strain (or combination of infections) predominates when a rebound epidemic occurs, this can lead to increased harm such as the recent epidemic of severe (sometimes fatal) viral hepatitis in children (Wise 2022).

Many of these harms could have been mitigated by implementing non-pharmaceutical interventions of less stringency or for shorter periods of time. While the use of less harmful interventions might in some cases need to be weighed against the potential benefits of such interventions, it should now be clear that the potential harms of pandemic policies were in many cases not adequately considered and could have been reduced by adopting alternative policies and/or relaxing harmful policies sooner rather than later.

## Proportionality

Proportionality is one of the most important ethical criteria for public health interventions. On the face of it, the requirement that public health measures are proportionate, i.e., that expected benefits must outweigh expected harms and burdens, seems straightforward. In practice, making assessments of proportionality can be complex not only because of (i) uncertain estimates of the probability and magnitude of different outcomes but also because (ii) different benefits and harms are incommensurable (i.e., because they are measured by different measures or are more or less quantifiable - consider, for example, weighing the benefit of averting a case of covid19 versus the harms of deteriorating mental health or disruption in a child’s life and education due to school closures), (iii) benefits and harms accrue to different individuals, and (iv) these outcomes occur at different times and in different places. Further, different value weightings will result in different ethical evaluations: individuals or communities that privilege avoiding harm to children will be more likely to eschew school closures, while those that prioritise individual liberty will have stronger reasons to reduce the use of restrictive policies and/or police enforcement. Yet there is presumably widespread agreement that, for example, it is worse to die as a child than to die as an older adult, or that curtailments of liberty that go beyond local social, legal, and political limits require strong ethical justification.

Any rigorous assessment of proportionality must begin with the acceptance that (i) all interventions are associated with both benefits and harms and (ii) the weightings of benefits and harms should not be arbitrary. For example, while avoiding cases of a viral respiratory disease is certainly a benefit, this benefit should not carry greater weight merely because it is highly salient in the context of a pandemic. Giving excessive weight to avoiding cases of a novel pathogen can result in disproportionate harms and ethically unacceptable sacrifices of other types of benefits (Lawford-Smith 2022). It is now clear that many NPIs instituted for covid19 were not proportionate, either because of minimal benefits or excessive harms. Examples of interventions with minimal or no evidence of significant benefits include all public health restrictions in well-ventilated outdoor spaces, the use of cloth masks, entry and exit screening (e.g. temperature checks), and the use of plastic screens and other similar interventions as (partial) barriers between normal human interactions. Examples of interventions with excessive harms include prolonged school and university closures; policies in hospitals, at borders, and in other settings that prevented human interaction at important moments (e.g., births, deaths, or during end-of-life care); extreme “hard lockdowns”, especially among marginalised populations (Silva 2020) and the effective ostracism of unvaccinated individuals from normal social interactions.

## Equity

One ethical goal of public health policy should be to ensure a fair distribution of the benefits and harms of interventions. Among other things, this means that it is arguably worse from a moral perspective if the harms of an intervention accrue to those who are already badly off (e.g., because of poverty, social marginalisation, etc.), especially if the benefits primarily accrue to those who are well off. Taken together, the response to covid19 has been profoundly inegalitarian, with many of the harms of lockdowns and mandatory policies being concentrated among the poor and marginalised, and many of the benefits being concentrated among the wealthy. Moreover, insofar as the goal of the pandemic response was often defined as reducing covid19 deaths, which occur primarily among older adults, there was a widespread inequitable bias in favour of older adults to the disadvantage of children and younger adults.

At the global and often at the local level, the response to covid19 resulted in a large increase in economic inequality (Oxfam 2022) – which, among other things, can be expected to increase health disparities between the rich and poor. These inequitable outcomes of policy choices are in stark contrast to claims that restrictive policies would promote equity. Early in the pandemic, some government agencies promoted the false claim that “this virus does not discriminate”; this might be taken to imply that all members of the population are equally at risk from the virus or that each person has similar reasons to comply with restrictive public health policies (Jamrozik and Heriot 2020). Yet, while a new coronavirus can and will eventually infect every living person, infections are often spread more easily in poor communities (e.g. due to crowded housing) and there are marked differences in the severity of infection depending on the average age and health status of those affected. In other words, the virus does discriminate - and equitable policy would take this into account by providing resources and focusing interventions where risks are highest, rather than imposing excessive restrictions where risks are low. Similarly, it is also untrue that in a pandemic “nobody is safe until everybody is safe”(Organization 2021). The outcomes of lockdowns showed that wealthy people who could work from home could be kept safe while essential workers and poor communities experienced the brunt of infections. While such claims might be superficially read as appeals to solidarity or equity, many public health policies adopted during the covid19 pandemic produced inequitable outcomes and exacerbated existing stark injustice in health outcomes.

## Reciprocity

Since many public health policies for infectious diseases involve harms and benefits that are unevenly distributed in populations, there are important ethical questions about what assistance and/or compensation is owed (and by whom) to those harmed or burdened by such policies(Holm 2020). One positive development has been the use of payments and other support for individuals subject to quarantine and isolation rules. Such support aligns with the principle of reciprocity in recognising that these rules aim to benefit others but typically involve a net reduction in wellbeing for the individuals who adhere to them.

The pandemic has also given rise to more complex questions regarding reciprocity. Given that covid19 is a much greater threat to older adults (and those with medical comorbidities), healthy children and young adults were asked (in many cases forced) to sacrifice a great deal of their own wellbeing over extended periods of time in order to reduce the probability of infection (and afford the time to develop vaccines) for those at higher risk of severe outcomes. At the international level, borders were closed, sometimes for prolonged periods, with the goal of reducing viral transmission from high incidence countries (often also low-income countries) to low incidence countries (often high-income and/or geographically isolated countries). Insofar as reciprocity carries ethical weight, one might think that greater compensation and support might be owed by the old to the young, and by wealthy “covid zero” countries to poor countries and/or to countries where vaccine field trials were conducted during epidemics (since such trials are impossible in countries where the pathogen is eliminated) (Heriot and Jamrozik 2021).

## Due legal process

While many pandemic public health policies were legal in the technical sense, this is often only because they took place in a state of emergency, when the norms of due legal process are suspended. In Australia and elsewhere, the legality of measures instituted using public health emergency powers often depended on whether a particular public health official considered that an intervention was justified. Officials were often under immense pressure and major decisions – including, for example, the “hard lockdown” of Melbourne public housing tower residents – were sometimes taken without due legal process and indeed without following considered public health advice(Glass 2020).

There was in many cases no meaningful judicial check on public health power. A legal challenge to nocturnal curfew in Melbourne, for example, was dismissed on the basis that the official who signed the curfew order considered it justified (rather than on the basis of whether or not it was actually justified by evidence that curfews would or did produce significant public health benefits exceeding their harms)(Supreme Court of Victoria 2020). While some jurisdictions have instituted reforms to address certain legal deficiencies in the democratic oversight of public health powers, prolonged states of emergency arguably undermine democracy itself, and there remains considerable scope for legal reform before the next pandemic.

## Transparency

In Australia and elsewhere, there was often minimal transparency regarding who was involved in key public health policy decisions, the details of such judgements, the advice provided to officials, and the evidentiary basis of public health advice. Politicians and public health officials often claimed to be “following the science”, without the details of that science or its interpretation by key officials being available for democratic scrutiny.

An early Australian ethics report suggested that transparency requires “openness about what decisions are being or have been made, for which reasons, and in accordance with what criteria” as well as “the disclosure of relevant interests, where applicable”(Council 2020). Similar requirements have been suggested in other jurisdictions, yet the pandemic revealed a lack of transparency even where decisions affected an entire population, often for prolonged periods of time. Many jurisdictions also appear to lack a standardised mechanism for making relevant conflicts of interest among health officials a matter of the public record.There is thus an opportunity to develop more transparent and accountable processes for evidence-informed decision-making during pandemics and other public health crises(Vickery, Atkinson et al. 2022).

## Conclusion

Considering many of responses to the Covid19 pandemic in light of the principles of public health ethics leads to some sobering conclusions. During the pandemic, the moral value of health often became narrowly aligned with the avoidance of one particular virus while mental health and other harms increased, socioeconomic inequalities were exacerbated, and civil liberties were subject to sometimes draconian limitations. The interests of children were in multiple ways sacrificed, often with no strong justification, in the name of reducing harm from a virus that poses extremely low risks to healthy children. Inequality skyrocketed; the benefits of public health interventions and their economic effects overwhelmingly accumulated to the rich while the poor benefited little, were often harmed, and were sometimes placed at higher risk of infection. There was a lack of evidence that the benefits of many NPIs outweighed their harms, and a widespread failure to collect such evidence in an unbiased way. Transparency and legal checks on power were often limited.

Policing was excessive. Rather than the “least restrictive alternative” populations experienced extreme levels of coercive control during lockdowns. Taken together, these failures risk undermining trust in public health and science, and the unchecked use of public health power (or prolonged states of emergency) risks undermining democracy itself.

Policies instituted during the covid19 pandemic provide many important case studies for ethical analysis. This Special Issue includes extended analyses of policies including lockdowns, school closures, border closures, and the use of fear in public health. It is hoped that better understanding of what went wrong, ethically speaking, during the last few years, might help to inform more balanced and proportionate responses to future pandemics.

## Footnotes

1 Here and elsewhere, I use “risk” in the sense of the probability of a harmful outcome such that risks are high insofar as the expected harm is high. This contrasts with (some) epidemiological uses of the term “risk” to refer to the probability of an outcome while being agnostic about the severity of expected harm.

2 “Woman in 70s contracted covid from fleeting exposure to Bondi limo driver” The Australian https://www.theaustralian.com.au/news/woman-in-70s-contracted-covid-from-fleeting-exposure-to-bondi-limo-driver/video/1ddf9ea0cc074f4fc192492f4e2e3324 [Accessed 1 August 2022].

3 https://www.covid19data.com.au/compare-lockdowns.

4 “WA government defends COVID breach punishments after 64 people jailed” Sydney Morning Herald 13 September 2021 https://www.smh.com.au/national/wa-government-defends-covid-breach-punishments-after-64-people-jailed-20210909-p58qdm.html [accessed 1 August 2022].
